# Supplementary material for: Preparation of Nickel Oxide Nanoflakes for Carrier Extraction and Transport in Perovskite Solar Cells
Source: Nanomaterials (Basel). 2022 Sep 25;12(19):3336. doi: 10.3390/nano12193336 (PMC9565255; doi:10.3390/nano12193336)
Supplement: Supplementary file 1 [file nanomaterials-12-03336-s001.zip › nanomaterials-1912767-supplementary.pdf]

Supporting Information

Preparation of Nickel Oxide Nanoflakes for Carrier Extraction and Transport in Perovskite Solar Cells

Chih-Yu Chang [1], You-Wei Wu [1], Sheng-Hsiung Yang [1,*] and Ibrahim Abdulhalim [2]

[1] Institute of Lighting and Energy Photonics, College of Photonics, National Yang Ming Chiao Tung University, No.301, Section 2, Gaofa 3rd Road, Guiren District, Tainan 71150, Taiwan
[2] Department of Electro-Optics and Photonics Engineering and the Ilse Katz Institute for Nanoscale Science and Technology, School of Electrical and Computer Engineering, Ben-Gurion University of the Negev, Beer-Sheva 8410501, Israel
* Correspondence: yangsh@nycu.edu.tw

Table S1. Lifetime parameters of TR-PL curves of the perovskite on the FTO substrate, PTAA, NiO_x thin film/PTAA, and NiO_x nanoflakes/PTAA.

Substrate	A_1 (%)	τ_1 (ns)	A_2 (%)	τ_2 (ns)	τ_{avg} (ns)
FTO	54.9	10.56	45.3	147.13	139.5
PTAA	50.69	16.07	49.31	103.02	91
NiO_x thin film/PTAA	51.29	16.51	48.71	83.9	72.34
NiO_x nanoflakes/PTAA	55.61	13.95	44.39	64.04	53.3

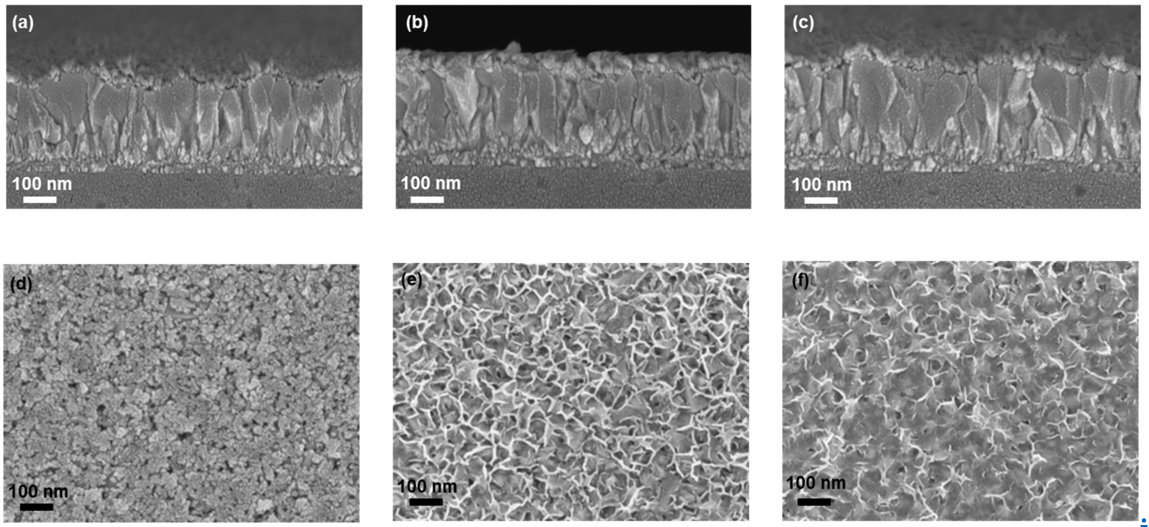

Figure S1. Cross-sectional and top-view SEM images of (**a**,**d**) ZnO nanorods, (**b**,**e**) mesoporous NiO_x before ZnO etching, and (**c**,**f**) NiO_x nanoflakes after ZnO etching.

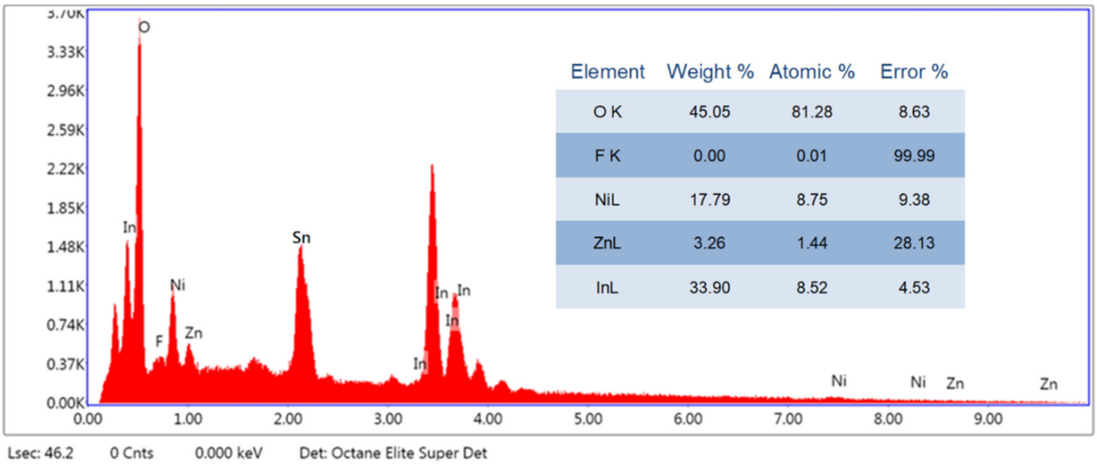

Element	Weight %	Atomic %	Error %
O K	45.05	81.28	8.63
F K	0.00	0.01	99.99
NiL	17.79	8.75	9.38
ZnL	3.26	1.44	28.13
InL	33.90	8.52	4.53

Figure S2. EDS spectrum of the NiO_x nanoflakes.

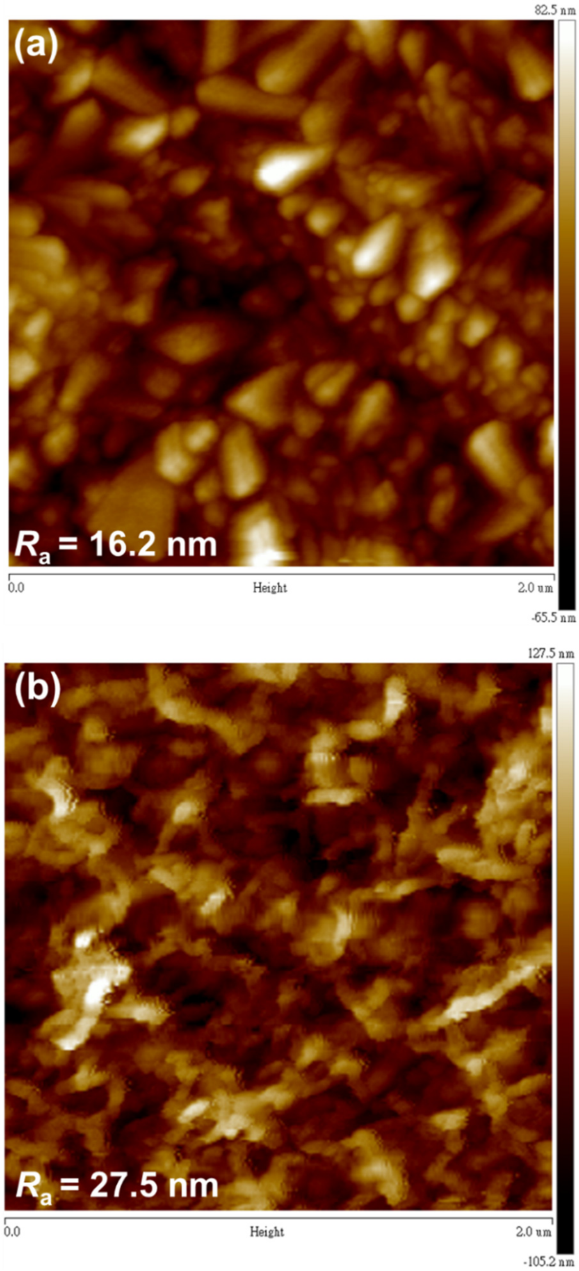

Figure S3. AFM topographic images of the NiO_x (**a**) thin film and (**b**) nanoflakes.

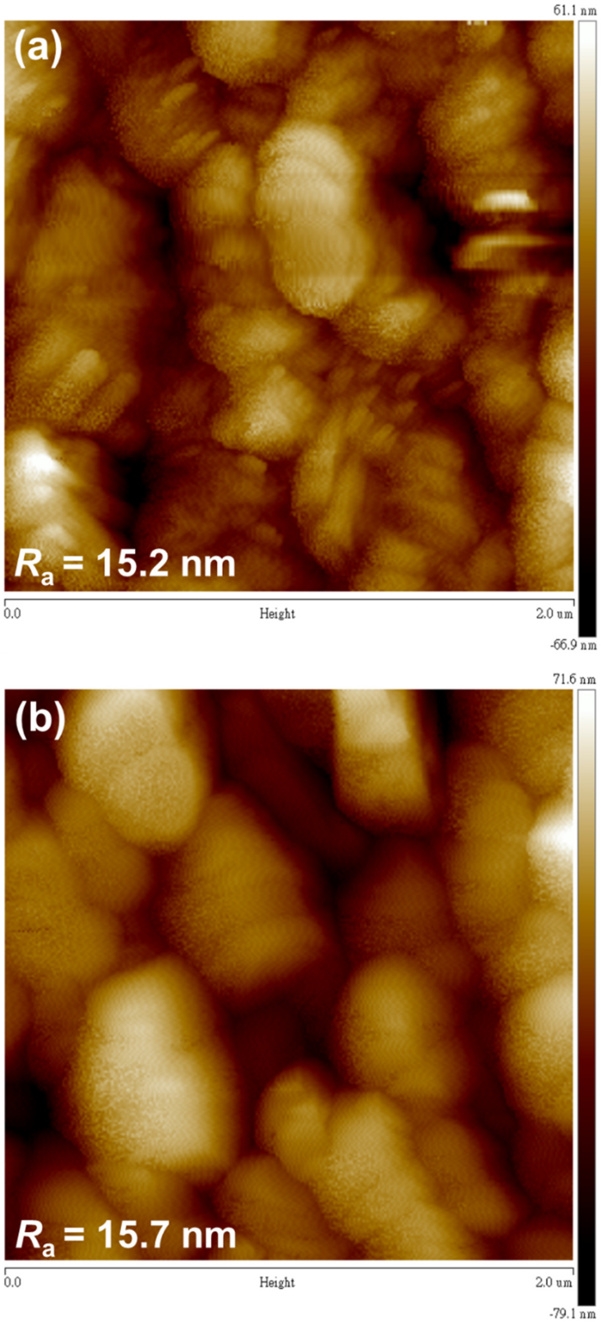

Figure S4. AFM topographic images of the perovskite on the (**a**) NiO_x thin film/PTAA and (**b**) NiO_x nanoflakes/PTAA.

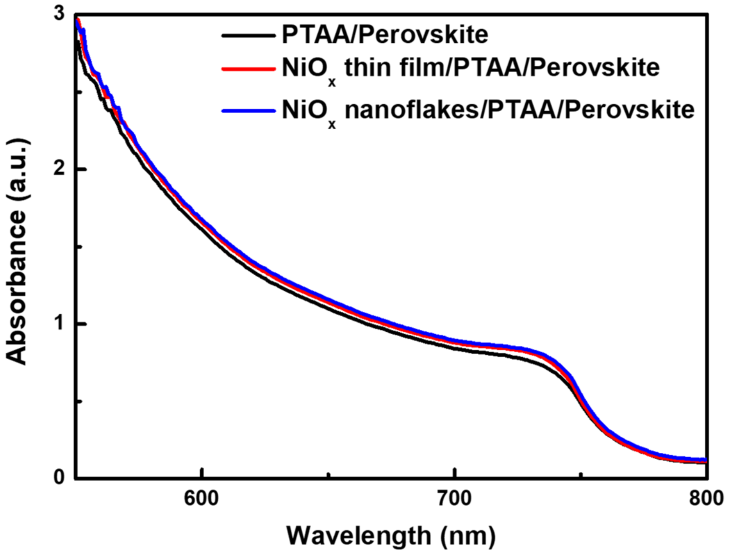

Figure S5. Absorption spectra of the perovskite on PTAA, NiO_x thin film/PTAA, and NiO_x nanoflakes/PTAA.

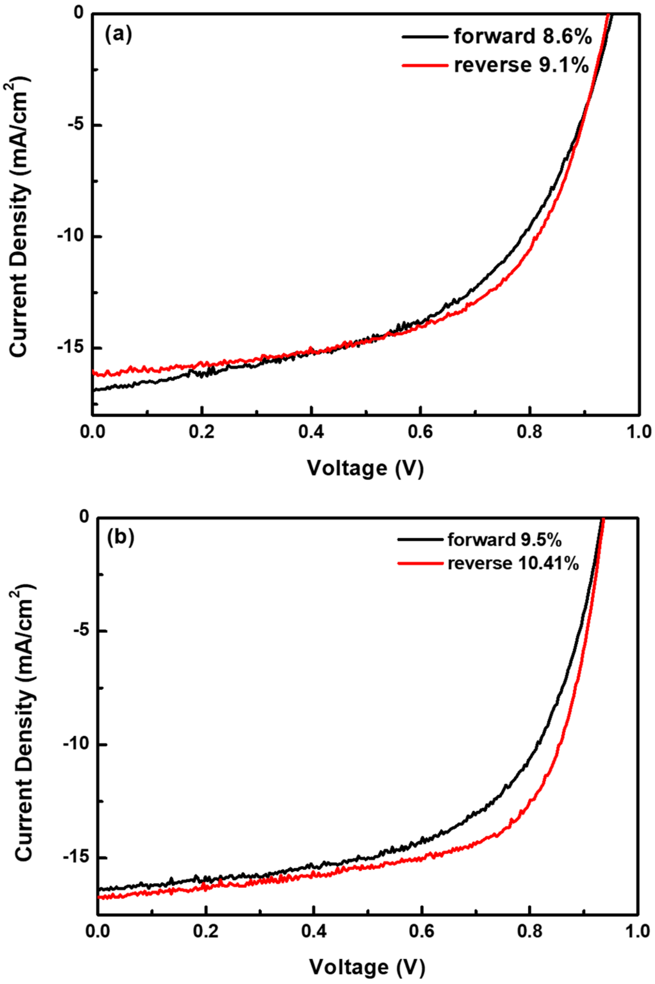

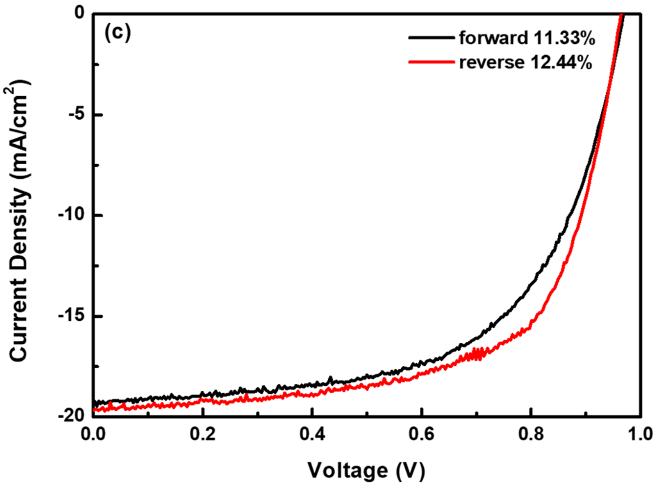

Figure S6. J–V characteristics and PCE values of the devices using (**a**) PTAA, (**b**) NiO_x thin film, and (**c**) NiO_x nanoflakes as the HTL.

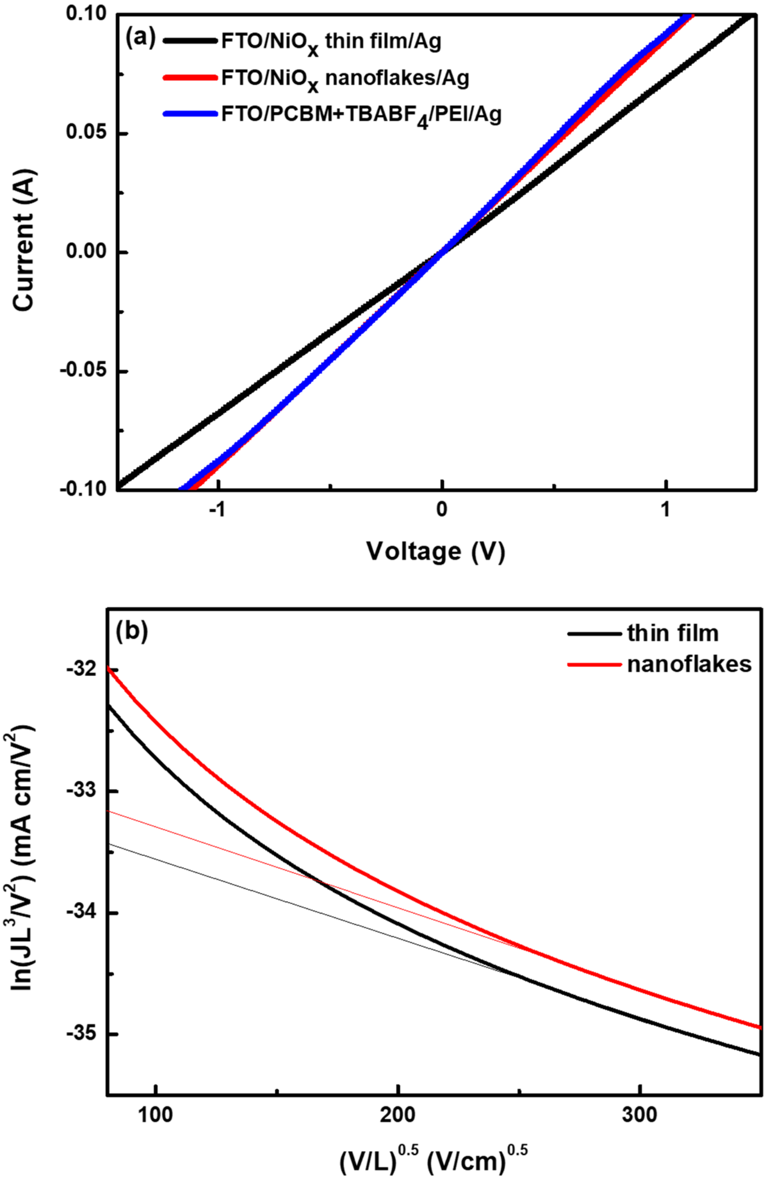

Figure S7. (**a**) Current–voltage characteristics of hole-only devices FTO/NiO_x thin film or nanoflakes/Ag and electron-only device FTO/PCBM+$TBABF_4$/PEI/Ag; (**b**) hole mobility of the NiO_x thin film or nanoflakes versus electric field $(V/L)^{0.5}$.

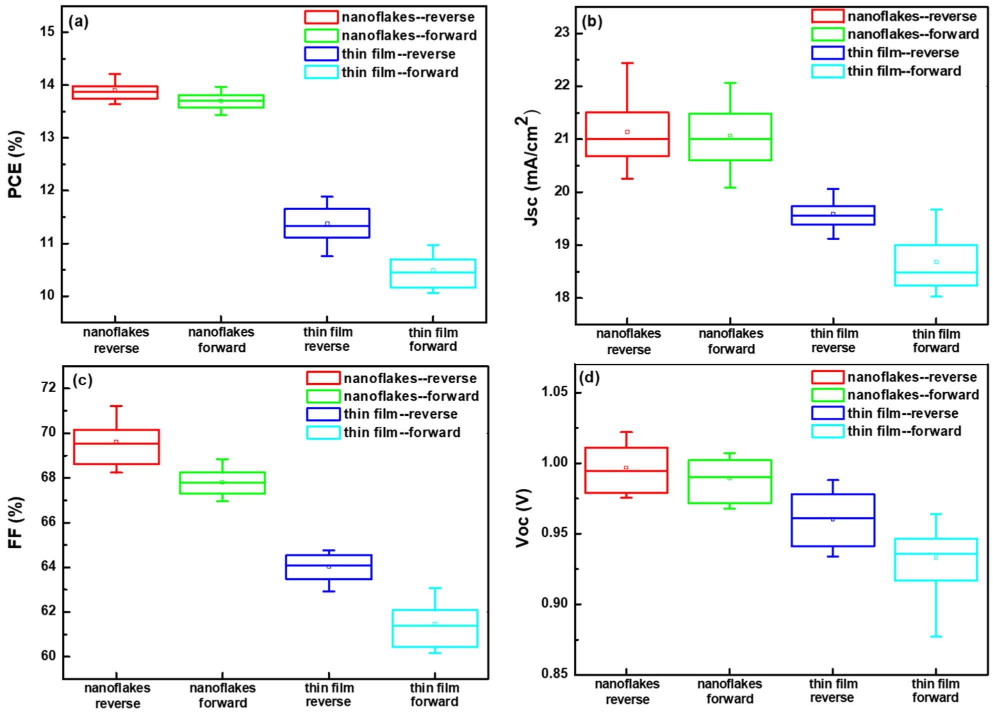

Figure S8. Performance variation represented as a standard box plot in (**a**) PCE, (**b**) J_{SC}, (**c**) FF, and (**d**) V_{OC} from 20 devices based on the NiO_x thin film and nanoflakes in the forward and reverse scans.
